# Characterization of APOBEC3G binding to 7SL RNA

**DOI:** 10.1186/1742-4690-5-54

**Published:** 2008-07-02

**Authors:** Daniel Bach, Shyam Peddi, Bastien Mangeat, Asvin Lakkaraju, Katharina Strub, Didier Trono

**Affiliations:** 1 Global Health Institute, School of Life Sciences and "Frontiers in Genetics", National Center for Competence in Research, Ecole Polytechnique Fédérale de Lausanne (EPFL), CH-1015, Lausanne, Switzerland; 2 Departments of Dermatology and Venerology, University Hospital, and of Microbiology and Molecular Medicine, Faculty of Medicine, University of Geneva, Geneva, Switzerland; 3 Department of Cell Biology, University of Geneva, CH-1211, Geneva, Switzerland

## Abstract

Human APOBEC3 proteins are editing enzymes that can interfere with the replication of exogenous retroviruses such as human immunodeficiency virus (HIV), hepadnaviruses such as hepatitis B virus (HBV), and with the retrotransposition of endogenous retroelements such as long-interspersed nuclear elements (LINE) and Alu. Here, we show that APOBEC3G, but not other APOBEC3 family members, binds 7SL RNA, the common ancestor of Alu RNAs that is specifically recruited into HIV virions. Our data further indicate that APOBEC3G recognizes 7SL RNA and Alu RNA by its common structure, the Alu domain, suggesting a mechanism for APOBEC3G- mediated inhibition of Alu retrotransposition. However, we also demonstrate that APOBEC3F and APOBEC3G are normally recruited into and inhibit the infectivity of ΔVif HIV1 virions when 7SLRNA is prevented from accessing particles by RNA interference against SRP14 or by over expression of SRP19, both components of the signal recognition particle. We thus conclude that 7SL RNA is not an essential mediator of the virion packaging of these antiviral cytidine deaminases.

## Background

APOBEC proteins are members of a family of polynucleotide cytidine deaminases (CDA) that play important roles in antiviral defence. Human APOBEC3G and 3F can block the replication of a wide array of exogenous retroelements, including retroviruses such as human immunodeficiency virus (HIV) and murine leukaemia virus (MLV) [1,2], and hepadnaviruses such as hepatitis B virus (HBV) [3,4]. Primate lentiviruses including HIV counter APOBEC3G and 3F via their Vif protein, which binds to and triggers the proteasomal degradation of these cellular antivirals. In the absence of Vif, APOBEC3G and -F are packaged into retroviral particles, and lethally edit nascent viral reverse transcripts [1,2,5,6]. What tethers APOBEC proteins to virions has so far remained incompletely characterized. While some have invoked a role for the viral genomic RNA, more undisputed is the claimed importance of the nucleocapsid region of HIV-1 Gag and of yet unknown cellular RNAs in this process [7-14].

APOBEC family members can also act on endogenous substrates, notably retroelements. APOBEC3A, 3G and 3F can block the propagation of endogenous retroviruses such as intracisternal-A particles (IAP) [15,16] or MusD, and APOBEC3A, 3B and, to a lesser extent, 3C and 3F can inhibit LINE-1 (Long Interspersed Nuclear Element 1) retrotransposition [15,17-20]. Furthermore, APOBEC3A, APOBEC3B, APOBEC3C and APOBEC3G can prevent Alu retrotransposition, a process mediated in trans by the reverse transcriptase and integrase activities encoded by LINE [17,18]. Interestingly, APOBEC3G overexpression appears to recruit Alu RNAs into APOBEC3G-containing high molecular mass ribonucleoprotein complexes [21].

The Alu family of repetitive sequences is one of the most successful groups of mobile genetic elements, having multiplied to more than one million copies in the human genome in some 65 million years of primate evolution [22,23]. Interestingly, the emergence of Alu as major primate genome remodelers has coincided with the expansion of the APOBEC3 gene family long before the appearance of modern lentiviruses [24,25], and there is a striking evolutionary coincidence between the expansion of the APOBEC gene cluster and the abrupt drop in retrotransposon activity that took place in primates, compared with rodents [26]. While the functions of Alu repetitive elements remain largely unknown, sequence analyses indicate that they originated from the evolutionary conserved 7SL RNA gene [27]. This gene encodes for the approximately 300-nucleotide-long RNA moiety of the signal recognition particle (SRP), a cytoplasmic ribonucleoprotein complex that associates with ribosomes to mediate the translocation of nascent proteins into the endoplasmic reticulum [28]. Interestingly, 7SL RNA was amongst the first host RNAs detected in avian and murine retroviral particles [29,30] and is packaged in HIV-1 virions at ten thousand and seven fold molar excess over the actin mRNA and viral genomic RNA respectively [31]. A recent study points to 7SL RNA as a mediator of APOBEC3G packaging into HIV virions [32]. The results of the present work rather support a model in which the interaction between 7SL RNA and APOBEC3G may shed light on APOBEC3G-mediated inhibition of Alu retrotransposition, but does not mediate the retroviral particle incorporation of the CDA.

## Methods

### Plasmids

Plasmids pCMV4-HA expressing the HA-tagged form of APOBEC3G and APOBEC3A were a kind gift from M. Malim (King's College, London, UK). Human APOBEC3B and APOBEC3F cDNAs were amplified from activated peripheral blood lymphocytes. We used primers cem196: 5'-agattagcttggctgaacatgaatccacagatcag-3' and cem197: 5'-ttacttctagagtttccctgattctggagaatgg-3' for APOBEC3B and primers cem157: 5'-agattaagcttccaaggatgaagcctcacttcag-3' and cem156: 5'-ttacttctagactcgagaatctcctgcagcttgc-3' for APOBEC3F. These cDNAs were then introduced in the HindIII and XbaI sites of the pCMV4-HA plasmid, replacing the human APOBEC3G cDNA. The resulting proteins correspond to the NP_004891 and NP_660341 NCBI entries, respectively. The cDNAs for APOBEC3C, APOBEC2, APOBEC1 and AID come from B. Matija Peterlin and Yong-Hui Zheng (University of California, San Francisco, USA) and were obtained through the NIH AIDS Research and Reference Reagent Program, Division of AIDS, NIAID, NIH. All cDNAs for APOBEC family members were inserted into the same expression vector pCMV4-HA. Single-aminoacid substitutions were made on APOBEC3G coding sequence using Quickchange site-directed mutagenesis kit (Stratagene) following the manufacturer's instructions. Plasmids for GAG expression and NC deletion construct, Zwt-p6, were kindly provided by P. Bieniasz (Aaron Diamond AIDS Research Center and the Rockefeller University, New York, USA). Plasmids for Alu retrotransposition, were a kind gift from T. Heidmann (Alu-Sb1: pAlu pA+ neoTet) and from J. Moran (L1-RP: pJM101 L1-RP Δ neo). Plasmids for SRP19 and SRP19 Δ 6 were kindly provided by Xiao-Fang Yu (Department of Molecular Microbiology and Immunology, Johns Hopkins Bloomberg School of Public Health, Baltimore USA). For 7SL RNA-APOBEC3G binding competition experiments, sequences encoding the 7SL Alu and S-stem domains were cloned in pLVCTH (ClaI-MluI sites; [33]) downstream of the pol-III promoter. Forward and reverse synthetic 60 nt nucleotides (Microsynth, Switzerland) were used to construct plasmids expressing shRNA against SRP14 and firefly luciferase in pSuper-Retro mammalian expression vector (Oligoengine inc). The target sequence for SRP14 is 5'-agggcatacatttcctgct-3' and as a control we used firefly luciferase 5'-cgtacgcggaatacttcga-3'.

### RNA structure analysis

Secondary structures of 7SL RNA and Alu-Sb1 were predicted using the Sfold software at the Sfold Web server (Wadsworth Center).

### Immunoprecipitations

HA-tagged APOBEC proteins and derivatives were expressed into Hela cells. In some cases competitors were co-transfected at the indicated ratios. 48 h later, total homogenate was obtained from confluent 10-cm plates using 500 μl per plate of high stringency RIPA lysis buffer (NP40 1%, Na Deoxycholate 0,5%, SDS 0,1%) complemented with 1× Protease Inhibitor Cocktail Set I (Calbiochem) and Prime RNase Inhibitor at 1000 units/ml (Eppendorf). Beads-immobilized anti-HA antibody (Roche) was used to immunoprecipitate HA-tagged proteins (50 μl beads + 200 μl homogenate), before extensive washes with low stringency lysis buffer and final elution with 100 μl of RNase-free distilled water.

### RNA detection and quantification

Eluted immunoprecipitates were used for reverse transcription using random hexanucleotide primers, and Superscript III reverse transcriptase (Invitrogen). Two sets of specific primers (5'-gcctgtagtcccagctactc-3', 5'-ccgaacttagtgcggacacc-3'; 5'atcgggtgtccgcactaag-3', 5'-gagtcctgcgtcgagagagc-3') were used to amplify 7SL RNA by SYBR-Green Real-Time PCR (Applied Biosystems). As an internal standard, 10^6^ copies of a lentiviral genomic plasmid  (pLVCTH, [34]) were included in each well, and viral genomic cDNA was amplified using specific primers (5'-ggagcagcaggaagcactatg-3', 5'-caggattcttgcctggagctg-3'; 5'ggagctagaacgattcgcagtta3', 5'ggtgtagctgtcccagtatttgtc3'). For endogenous Alu RNA amplification the following primers were used: 5'-cactttgggaggccgaggcg-3' and 5'-gtagctgggactacaggcgc-3'.

### ALU retrotransposition assay

pAlu pA+ neoTet (1 μg), pJM101 L1-RP Δneo (1 μg), and cytidine deaminases-expressing plasmids (1 μg) were co-transfected into 10^5 ^HeLa cells using JetPei (Polyplus). The day after they were plated on 10 cm dishes, and selected once they had reached confluence in 2 mg/ml G418 (Invitrogen). After 60 h medium was changed and G418 concentration was reduced to 0,5 mg/ml. After 72 h colonies were fixed and coloured (20% methanol, 1 g/l crystal violet).

### Virological assays

**Figure 1:**
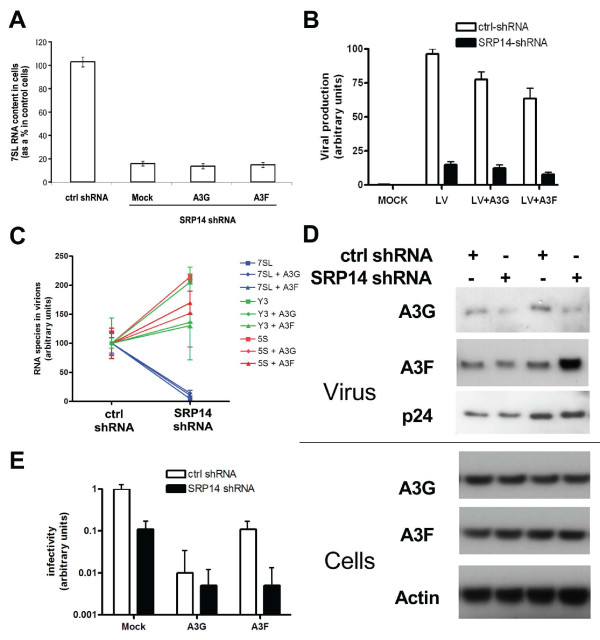
**7SL RNA knockdown does not prevent A3G encapsidation**. A. 7SL RNA in cells lines expressing control-shRNA and SRP14-shRNA, was quantified by real time PCR. APOBEC3G or APOBEC3F expressing plasmids were transfected when indicated. Means and standard errors from three independent experiments are shown. B. Production of Vif-defective HIV-1 particles from control-shRNA or SRP14-shRNA cell lines, in the presence of APOBEC3G or APOBEC3F when indicated. Means and standard errors from three independent experiments are shown. C. 7SL RNA, Y3 RNA and 5S RNA measured by real time PCR in Vif defective HIV-1 particles produced from control-shRNA or SRP14-shRNA cell lines, upon transfection of APOBEC3G or APOBEC3F when indicated. Results were normalized to viral genomic RNA. Means and standard errors from three independent experiments are shown. D. Western blot analysis of these viruses and of cytoplasmic extracts of the producer cells, using indicated antibodies, ß-actin and capsid serving as loading controls. E. Infectivity of Vif-defective HIV-1 particles produced from control-shRNA or SRP14-shRNA cell lines, in the presence of APOBEC3G or APOBEC3F when indicated.

Vif-defective HIV-1 particles were produced by transient transfection of 293T cells with Fugene (Roche) in presence or absence of antiviral cytidine deaminases with or without HIV-1 Vif. In some cases (as in figure 1) shRNA-expressing plasmids were cotransfected. 1 ml of supernatant was then spun in 1,5 ml eppendorf tubes at 13'000 rpm in a microfuge at 4° for 90 min. Pellets were resuspended in PBS 1% Triton, and particles were quantified by a standard RT assay, measuring relative infectivity by titration on CD4+, LTR-LacZ-containing, HeLa-derived P4.2 cells. Normalized amount of virions were then loaded on standard Laemmli protein gels to perform Western blots. 

APOBEC3G-specific immunofluorescence was performed as previously described (25).

### 7SL RNA knockdown

293T cells were seeded at 60% confluency in 6 cm plates in triplicates and transfected with pSR14 and pSR-Luc, which produces shRNA against SRP14 subunit and Luciferase respectively, using calcium phosphate mediated transfection. Puromycin dihydrochloride (3 μg/ml, Sigma) was used to select the transfected cells 24 hours post-transfection. After 24 hours more, cells were washed with TBS (Tris buffered saline) and replaced with fresh medium containing puromycin at 0.5 μg/ml. At 120 hours after transfection the cells expressing shRNA were further transfected with lentiviral plasmids together with APOBEC3G and APOBEC3F. Cells and virions were collected at 144 hours after transfection when the SRP14 and 7SL RNA are significantly downregulated. Cells were grown in the presence of cycloheximide (Sigma) during the whole procedure at 5 μg/ml concentration to improve efficiency of targeting into ER and thereby improving the viral titer.

### 7SL RNA down regulation by SRP19 over expression

7 × 10^6 ^293T cells were seeded into 15 cm plates in duplicates approximately 24 h before transfection with HIV-1 ΔVif and SRP19myc or SRP19 Δ6myc plasmids at ratios of 4:1 and 2:1 (SRP19:HIV-1), in presence and absence of A3G and A3F. Virus was collected 36 h post medium change. Virus supernatant was cleared of cellular debris by centrifugation at 3000 rpm for 15 min in Heraeus megafuge centrifuge and filtration through a 0.2-μm pore size membrane (Millipore). Virus particles were then concentrated without sucrose cushion by ultracentrifugation at 196,000 × g for 2 h at 16°C in a Beckmann Coulter optima L-80 XP ultracentrifuge. Viral pellets were suspended in lysis buffer [(PBS-containing 1% Triton X-100, 1× Protease Inhibitor Cocktail Set I (Calbiochem) and RNase inhibitor (Promega)]. Cellular and viral RNAs were extracted using RNeasy Micro Kit (Qiagen, 74004) and QiAMP Viral RNA mini kit (Qiagen, 52904), respectively.

### Immunoblot analyses

Normalized quantities of samples from both cells and virus were suspended in 5× sample buffer and 20× reducing agent (Fermantas) denatured for 5' at 95°C and resolved on a Tris-glycine SDS-Polyacrylamide gel followed by western blot. HA-tagged proteins (A3G and A3F) were detected using peroxydase-conjugated rat monoclonal antibody (clone 3F10, Roche). Proliferating cell nuclear antigen (PCNA) was used as a protein loading control and was detected using a mouse monoclonal antibody (clone PC10, Calbiochem) followed by a secondary sheep anti-mouse antibody conjugated to horseradish peroxidase. Myc tagged proteins (SRP19 and SRP19Δ6) were detected using C-myc rabbit polyclonal antibody (SC-789) from Santacruz followed by secondary donkey anti-rabbit antibody conjugated to horseradish peroxidase. p24 (Capsid) of virus was detected using anti p24 antibody (mouse, from AIDS reagent programme) followed by secondary sheep anti-mouse antibody conjugated to horseradish peroxidase.

## Results

### Binding of APOBEC3G to 7SL RNA

**Figure 2:**
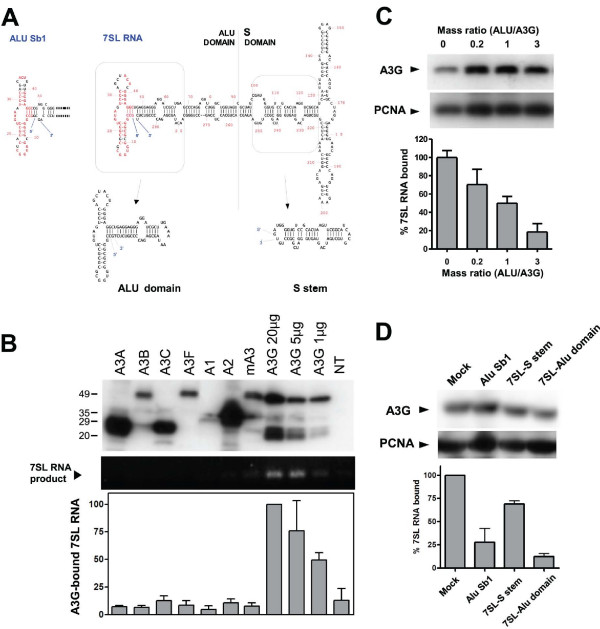
**APOBEC3G binds 7SL RNA**. A. Predicted secondary structure of Alu and 7SL RNAs. The latter comprises Alu and S domains. Bases in red are identical between the Alu used in this study (belonging to Alu Sb1 family) and 7SL. For competition experiments (see 2D), the indicated Alu and S stem domains of 7SL RNA were used. B. Indicated HA-tagged cytidine deaminases were immunoprecipitated. The resulting material was analyzed by HA-specific western blot (top) and 7SL RNA-specific standard (middle) or real-time (bottom) RT-PCR. For APOBEC3G, indicated three doses of plasmid were transfected; for all the others, 20 μg of DNA were used. NT, non-transfected. C. Alu RNA competes with 7SL RNA for binding to APOBEC3G. Increasing doses of Alu-Sb1 RNA-expressing plasmid were co-transfected with a fixed amount of APOBEC3G DNA. Upper panel: APOBEC3G in total cellular homogenates in one representative experiment. PCNA was blotted as a loading control. Bottom panel: APOBEC3G was immunoprecipitated and Real Time PCR was used to quantify levels of 7SL RNA in the immunoprecipitates. Means and SE from three independent experiments are shown. D. The Alu domain of 7SL RNA competes with full-length 7SL RNA for APOBEC3G binding. Alu-Sb1 RNA or 7SL Alu and S stem domains-expressing plasmids were co-transfected with APOBEC3G DNA at a 3:1 ratio. Upper panel: APOBEC3G in total cellular homogenates in one representative experiment, PCNA serving as a loading control. Bottom panel: 7SL RNA-specific real time PCR on APOBEC3G-specific immunoprecipitates. Means and SD from two independent experiments are shown.

The Alu and 7SL RNAs are closely related and share a common secondary structure: the Alu domain (Fig. 2A). Accordingly, the demonstrated interaction between APOBEC3G and Alu RNA [21], and the specific incorporation of 7SL RNA in HIV-1 particles [31] suggested that 7SL RNA might bind APOBEC3G, and perhaps mediate the recruitment of the cytidine deaminase into virions. To probe this issue, we first immunoprecipitated extracts of HeLa cells expressing HA-tagged APOBEC proteins with a HA-specific antibody, and subjected the resulting material to Western blotting and 7SL RNA-specific RT-PCR (Fig. 2B). 7SL RNA was detected in human APOBEC3G-specific immunoprecipitates, but not in association with APOBEC1, 2, 3A, 3B, 3C or 3F, nor with murine APOBEC3. By transfecting serial dilutions of the APOBEC3G-HA plasmid, we confirmed that 7SL RNA recovery was proportional to the levels of immunoprecipitated protein, and that the failure to detect this RNA in association with other APOBEC family members was not due to less efficient recovery of these latter proteins. Moreover, overexpression of an Alu RNA (Alu-Sb1) inhibited the recruitment of 7SL RNA by APOBEC3G (Fig. 2C), consistent with a model in which the cytidine deaminase recognizes the Alu domain of the SRP RNA constituent. In order to confirm this result, we overexpressed the 7SL Alu and S-stem domains (Fig. 2A) and tested their effect in similar competition experiments. We found that the 7SL S-stem domain had little effect on APOBEC3G binding to full-length 7SL RNA, whereas the 7SL Alu domain strongly interfered with this interaction, even more effectively than Alu-Sb1 (Fig. 2D). This suggests that APOBEC3G binds to the Alu domain of 7SL RNA.

### W127 of APOBEC3G is essential for Alu inhibition, 7SL and Alu RNAs binding and packaging into HIV virions

**Figure 3:**
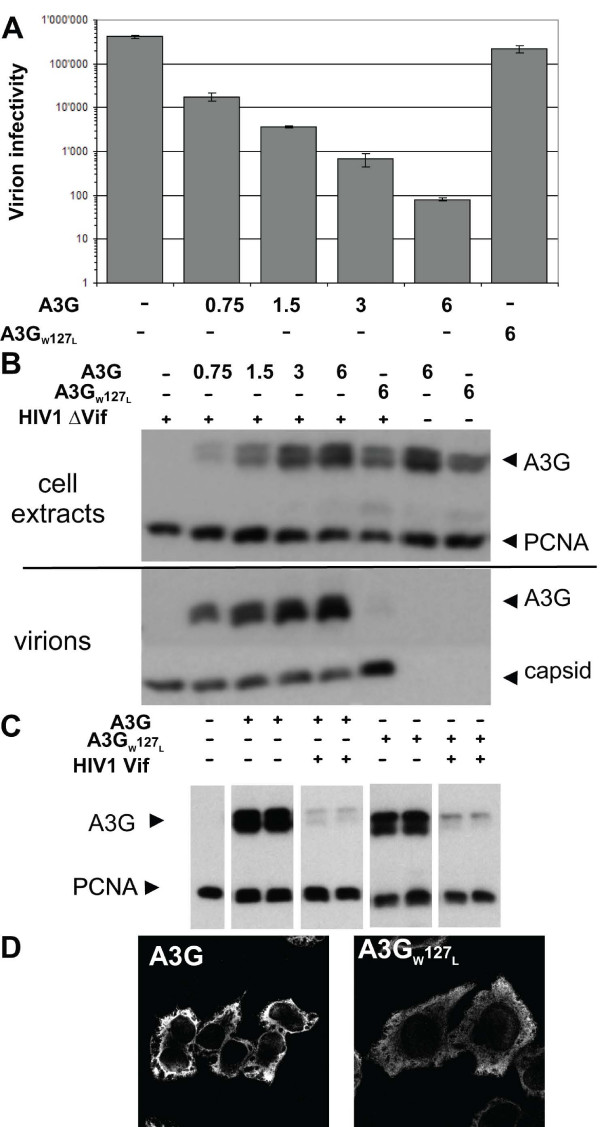
**A packaging-defective APOBEC3G mutant**. A. Infectivity of Vif-defective HIV-1 particles (expressed in HeLa P4.2 transducing units normalized for RT activity) produced in the presence of wild-type (A3G) or W127L APOBEC3G by transfection of 293T cells. Numbers below columns represent amount of transfected DNA in μg. Representative of five independent experiments. B. Western blot analysis of these particles and of cytoplasmic extracts of the transfected cells, using indicated antibodies, PCNA and capsid serving as loading controls. Although well expressed, APOBEC3GW127L is not packaged into virions. C. APOBEC3GW127L is sensitive to HIV-1 Vif-induced degradation. HIV-1 Vif was co-expressed in 293T cells with either wild type or W127L HA-tagged APOBEC3G, and extracts were analyzed by HA-specific Western blotting. D. HA-specific indirect immunofluorescence analysis of cells expressing HA-tagged versions of wild-type or W127L APOBEC3G. Both proteins localize to the cytoplasm, although the W127L mutant may exhibit a slightly more diffuse distribution.

**Figure 4:**
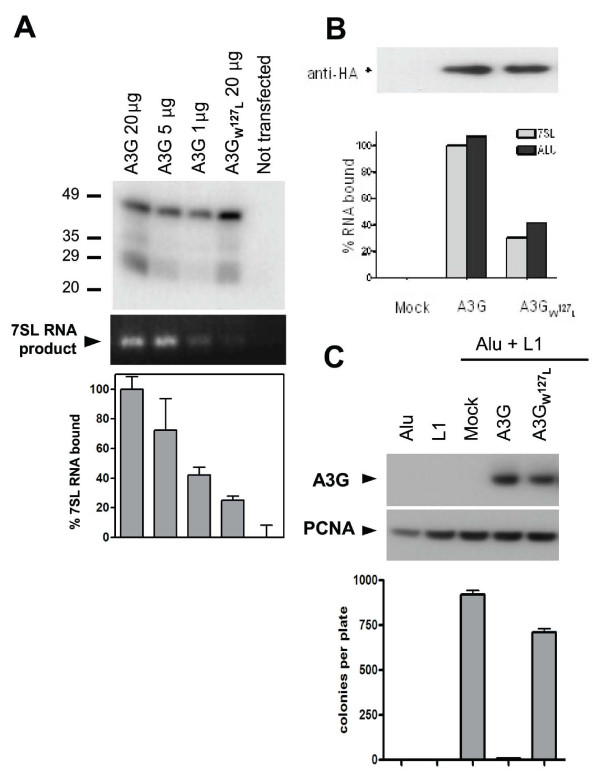
**APOBEC3G packaging mutant is defective for 7SL and Alu RNAs binding, and it does not inhibit Alu retrotransposition**. A. 7SL RNA capture assay as described in figure 1B, transfecting 293T cells with indicated doses of wild-type or W127L APOBEC3G-expresssing plasmid. Top: HA-specific Western blot; middle: standard RT-PCR; bottom: real-time RT-PCR. B. 7SL RNA and endogenous Alu RNAs capture assay as described in figure 1B, transfecting 293T cells with indicated doses of wild-type or W127L APOBEC3G-expresssing plasmid. Top: HA-specific Western blot; bottom: real-time RT-PCR. C. Alu (pAlu pA+ neoTet) and L1 (pJ M101 L1-RP Δneo) expressing plasmids were transfected in HeLa cells in the presence of the indicated cytidine deaminases. Western blot analysis of total cell extracts, with PCNA- and HA-specific antibodies (top). Scoring of G418-resistant colonies (bottom). Means and SE from three representative experiments are shown.

To investigate a hypothetical role for Alu-related RNAs in APOBEC3G HIV-1 virion incorporation, we turned to a library of point mutants of the cytidine deaminase. We identified a series of single amino acid mutants with either partial (H65R, W94L, C97S, Y124A) or complete (Y91A, R122A, W127L) HIV packaging defect, which correlated with an inability to block the infectivity of Δ Vif HIV-1 (not illustrated). Amongst the three mutants that completely failed to be incorporated in HIV-1 virions, W127L stood out as exhibiting the same stead-stated levels of expression and cytoplasmic localization as wild type, and moreover was fully sensitive to Vif-induced degradation (Fig. 3). We thus decided to concentrate on this mutant, which had also a markedly reduced ability to bind 7SL RNA (Fig. 4A). In agreement with our previous finding that 7SL RNA binding involves recognition of 7SL Alu domain, we could PCR amplify endogenous Alu RNA from wild-type but not W127L APOBEC3G immunoprecipitates (Fig. 4B). Accordingly, this mutant was not able to block Alu retrotransposition (Fig. 4C).

### A NC-deleted HIV-1 Gag mutant fails to package both APOBEC3G and 7SL RNA

**Figure 5:**
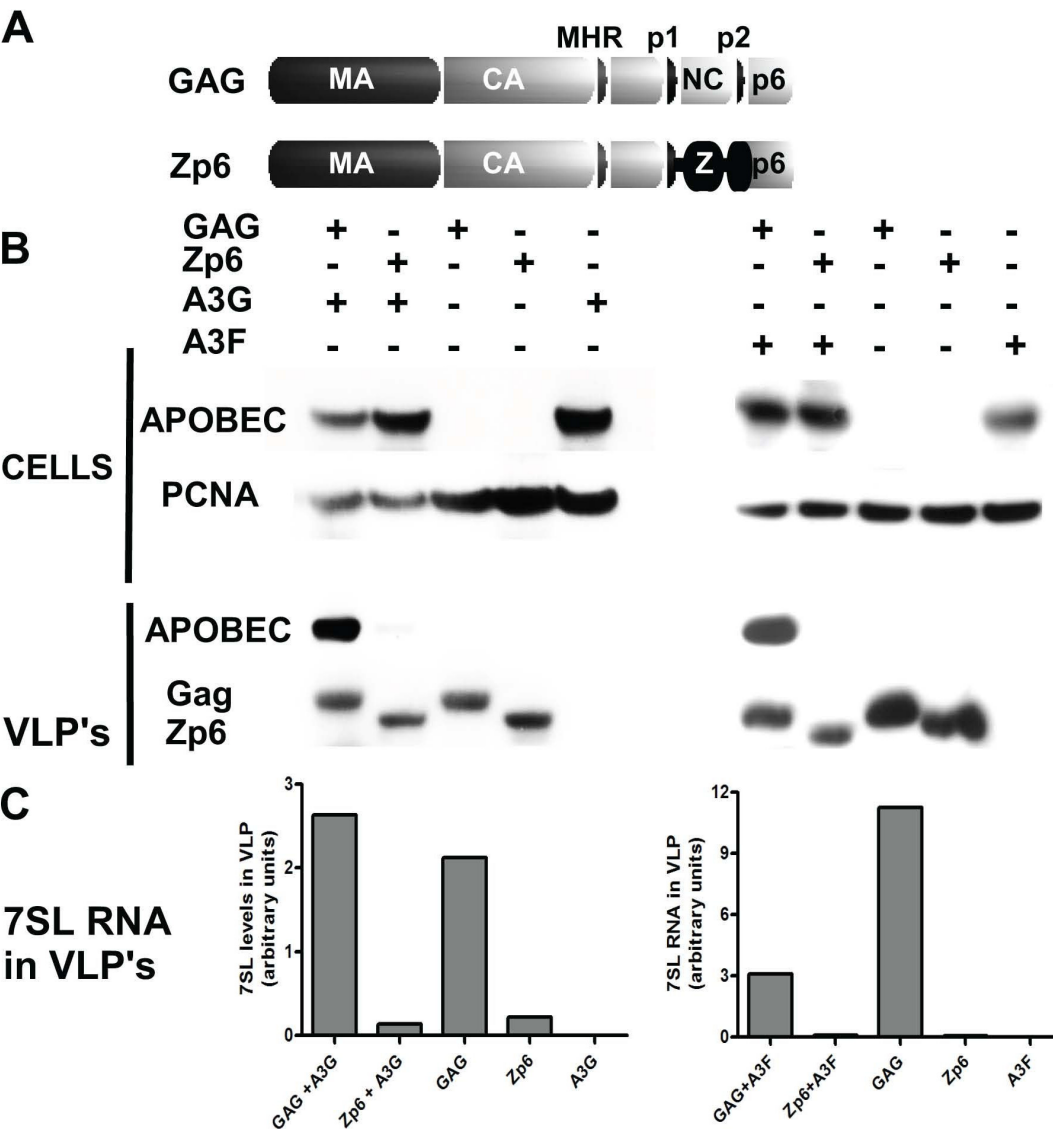
**7SL RNA packaging into HIV-1 virus like particles is dependent on nucleocapsid**. A. Schematic representation of HIV-1 VLP-forming GAG construct and its derivative, Zwt-p6. B. VLPs were produced by 293T cells transfection with GAG or Zwt-p6 constructs in the presence of APOBEC3G, APOBEC3F or mock plasmid as indicated. Intracellular and VLP levels of cytidine deaminases and Gag were measured by Western blotting with HA- and capsid-specific antibodies, respectively. C. 7SL RNA in viral like particles was quantified by real time PCR. A representative experiment out of two is shown.

Several studies have pointed to the role of some cellular RNA(s) as a bridge between NC and APOBEC3G, important for the virion packaging of the CDA [7-14,35,36]. HIV-1 viral-like particles (VLP) can be produced from a Gag derivative in which NC is replaced by a heterologous sequence providing the intermolecular Gag interaction function normally fulfilled by this region. It was previously demonstrated that one such chimerical protein termed Zwt-p6, in which NC is replaced by a leucine zipper from GCN4 (Fig. 5A), induces the efficient formation of virions, but that these contain low levels of non viral RNA and fail to incorporate APOBEC3G [14]. We indeed found that Zwt-p6 VLPs contained about ten times less 7SL RNA than wild-type Gag VLPs, as recently reported [32] and very low levels of not only APOBEC3G but also APOBEC3F (Fig. 5BC).

### HIV-1 virions devoid of 7SL RNA still contain and are normally inhibited by APOBEC3F and APOBEC3G

These results were consistent with a model in which 7SL RNA mediates the recruitment of APOBEC3G into HIV-1 virions, although they provided only correlative evidence. To probe the issue more directly, we examined the incorporation of the cytidine deaminase into virions produced from cells in which the SRP RNA was downregulated. First we tried to downregulate 7SL RNA by transfection of small interfering RNAs targeted against its sequence. However, these attempts were unsuccessful (data not shown). We thus turned to an indirect approach. For this, we took advantage of the fact that downregulation of SRP14 (a protein constituent of the SRP) by RNA interference leads to destabilization of the signal recognition particle and degradation of 7SL RNA (Fig. 1A). It was previously demonstrated that cell growth and protein translocation defects caused by low levels of functional SRP can be prevented by slowing down nascent chain elongation with sublethal doses of the protein synthesis inhibitor cycloheximide, which restore the secretion pathway [37]. We thus produced HIV-derived lentiviral vector particles in the presence of either APOBEC3G or APOBEC3F from 293T cells expressing SRP14-shRNA or control-shRNA, using this protocol. Viral production was diminished five fold when 7SL RNA was downregulated, whether or not a cytidine deaminase was present (Fig. 1B). Virions produced from SRP14-shRNA expressing cells contained about 100-fold less 7SL RNA than control, as measured by quantitative PCR, normalizing for the viral genomic RNA. In contrast, levels of other small cellular RNA species previously shown to be incorporated in HIV virions, such as Y3 and 5S, were either unchanged or augmented (Fig. 1C). In spite of the marked drop in 7SL RNA, intraviral concentrations of APOBEC3G were only very slightly diminished, and those of APOBEC3F actually increased (Fig. 1D). From these data, we conclude that the SRP scaffold RNA is not essential for the virion incorporation of these cytidine deaminases. Of note, virions lacking 7SL RNA exhibited significantly reduced levels of infectivity (Fig. 1E), suggesting that the selective packaging of 7SLRNA into HIV-1 virions could be functionally relevant. However, we cannot exclude non-specific consequences of the manipulation of the protein production machinery inherent to our approach. Importantly, however, the inhibitory effect of A3F and A3G was still fully obtained on 7SL RNA-less particles (Fig. 1E).

**Figure 6:**
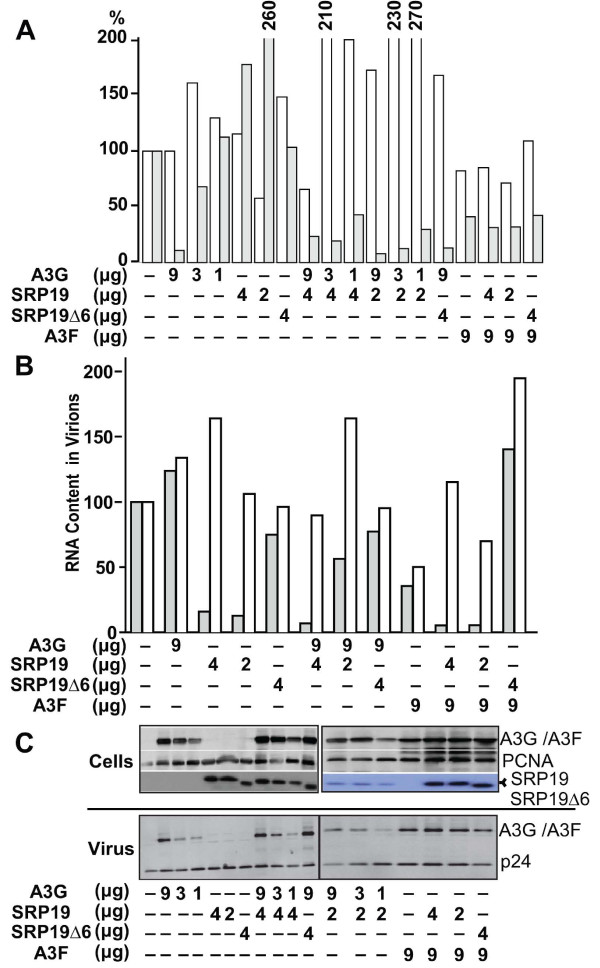
**7SL RNA down regulation by over expression of SRP19 does not inhibit APOBEC3F and 3G encapsidation and antiviral effect**. A. Effect of SRP19 overexpression on virus production and infectivity. HIV-1 ΔVif lentiviral vector particles were produced by transient transfection of 293T cells, adding indicated amounts of APOBEC3G, 3F, SRP19 and SRP19 Δ6 plasmids. Virion production (white columns) and infectivity (gray columns) were assessed by measuring reverse transcriptase activity in the supernatant and performing a single round transduction assay, respectively. SRP19 overexpression affects neither HIV particle release nor the antiviral action of APOBEC3G and 3F. Results are representative of three independent experiments. B. SRP19 over expression inhibits 7SL RNA virion incorporation. HIV genomic (white columns) and 7SL RNA (gray columns) levels in virions produced as described in (A) were quantified by real time PCR. Amounts measured in virions produced from cells transfected with ΔVif HIV1 alone were given the arbitrary value of 100. SRP19 overexpression drastically reduces 7SL RNA levels in virions, in spite of having no measurable effect on the intracellular levels of this RNA species (not illustrated). Representative of three independent experiments. C. SRP19 overexpression does not impair APOBEC3F/3G virion incorporation. Western blot analysis of viral and cellular extracts after transient transfection of 293T cells as described in (A), using indicated antibodies. The normal virion incorporation of A3G and A3F corroborates the absence of effect of SRP19 overexpression on the antiviral action of the cytidine deaminases (A).

To confirm these data, we used a second approach. SRP19 is a key component in the formation of the SRP complex, demonstrated to be the first SRP protein to bind the 7SL RNA during assembly of the complex [38], and to induce conformational changes in this RNA [39]. It was recently suggested that sequesteration of 7SL RNA by over expression of SRP19 decreases APOBEC3G [32] as well as APOBEC3F [40] packaging into HIV1 virions. Since this result was at odd with our own data obtained by SRP14/7SL RNA downregulation (Fig. 1), we produced ΔVif HIV1 virions in cells overexpressing SRP19 (at SRP19: HIV plasmid ratios of 4:1 or 2:1) in absence or presence of APOBEC3F or APOBEC3G. Under all the conditions, viral production was normal (Fig. 6A), contrasting with what observed in case of SRP14 knockdown (Fig. 1B). While without impact on cellular levels of 7SL RNA (not illustrated), SRP19 over expression lead to a more than 90% decrease in the virion incorporation of this RNA species (Fig. 6B). In spite of this effect, and contrary to the results of recent studies [32,40], there was no corresponding decrease in the packaging of APOBEC3G or APOBEC3F into virus particles (Fig. 6C). Moreover, the inhibitory effect of the cytidine deaminases on the infectivity of ΔVif HIV1 was at least as pronounced upon SRP19 over expression (Fig 6A). Overall, these results demonstrate that 7SL RNA is not responsible for mediating packaging of neither APOBEC3G nor APOBEC3F.

## Discussion

In this work we characterize the binding of APOBEC3G to 7SL RNA, the nucleic acid scaffold of the signal recognition particle (SRP). The SRP contains, in addition to 7SL RNA (or SRP RNA), at least six polypeptides, named according to their apparent molecular mass (SRP9, 14, 19, 54, 68 and 72). This multimolecular complex recognizes the signal peptide present at the N-terminal end of secreted and transmembrane proteins, as it emerges from ribosomes translating the corresponding mRNAs. These mRNAs are then targeted by the SRP to the endoplasmic reticulum (ER) membrane, where translation of the new ER-translocated protein continues. Assembly of the SRP proceeds through the recognition of the 7SL RNA Alu domain by the SRP9-SRP14 heterodimer [41], the other SRP proteins, including the SRP68-SRP72 heterodimer, binding the 7SL RNA S domain (reviewed in [42]. SRP9 and SRP14 also bind Alu RNAs by interacting with their so-called Alu domain [28], the common signature of all Alu-related RNAs. Noteworthy SRP9 and SRP14 and Alu RNAs have been recently identified as part of an APOBEC3G-containing high molecular weight complex [21] Alu emerged during primate evolution by deletion of the S domain of the 7SL RNA, yielding elements that rapidly spread in the primates genome via reverse transcription and integration through the action of proteins encoded by LINE [43]. APOBEC3A, 3B, 3C inhibit LINE-1 retrotransposition [15,17,19,20], and concomitantly blocks LINE-mediated Alu retrotransposition [17]. Less clear is the role of APOBEC3F, which has been shown to inhibit LINE-1 retrotransposition in some [15,19,20], but not all studies [17,18], and to have only a mild effect on Alu retrotransposition [18]. Strikingly, APOBEC3G is able to inhibit LINE-mediated Alu retrotransposition [18,21] despite being largely inactive on LINE-1 retrotransposition [15,17-19,21]. Here, we characterize APOBEC3G specific binding to the Alu-related 7SL RNA, and demonstrate that this interaction can be competitively inhibited by overexpressing the Alu-Sb1 RNA or the Alu domain of 7SL RNA. Furthermore, the study of a collection of APOBEC3G single amino acid mutants identified a derivative, carrying a tryptophan to leucine change in the first zinc-coordinating motif (APOBEC3G_W127L_), that was stably expressed, W127L properly localized, and still sensitive to Vif-induced degradation, but defective for 7SL RNA and Alu RNA binding. This mutant also failed to inhibit Alu retrotransposition. These data support a model whereby APOBEC3G blocks Alu retrotransposition by recognizing the Alu domain common to all Alu-related RNAs, rather than the trans-acting LINE-encoded Orf2 protein. This is consistent with recent evidence suggesting that APOBEC3G does not interact with any of the polypeptides encoded by LINE-1 [18] and, when overexpressed, can recruit Alu to a high molecular mass nucleoprotein complex [21]. The binding of APOBEC3G to the 7SL and Alu RNAs could be direct, or via the SRP9/14 heterodimer. In contrast, it is possible that APOBEC3A, 3B, 3C and 3F inhibit Alu retrotransposition via their effect on LINE since none of these proteins was found here to bind the Alu-related 7SL RNA.

7SL RNA is the most abundant RNA found in retroviral particles. For each molecule of viral genomic RNA, there are 7 molecules of 7SL RNA in an HIV-1 virion, and levels of 7SL RNA in particles are ten-thousand fold higher than those of actin messenger RNA, even though the latter is much more abundant in producer cells [31]. 7SL RNA thus appears to be actively incorporated into nascent particles during retroviral assembly. Cumulated evidence demonstrates that the nucleocapsid (NC) region of HIV-1 Gag mediates the recruitment of cellular RNAs into virions [35,44]. Here, when we produced VLPs with NC-deleted versions of HIV-1 Gag, virion levels of 7SL RNA were decreased about ten fold, and APOBEC3G and APOBEC3F also failed to be incorporated. These results confirm previous work indicating that APOBEC3G packaging into HIV virions is an RNA-dependent process requiring the NC basic linker [8,14,32] and extend this finding to APOBEC3F, but contradict the suggestion that 7SL RNA packaging is independent of NC [31,45]. We cannot explain this discrepancy, although we feel that the highly quantitative nature of our real-time RT-PCR measurements may provide a more rigorous assessment than the techniques used in these two other studies, namely an RNase protection assay with a probe recognizing at the same time the HIV-1 genomic and the 7SL RNA or a non-quantitative RT-PCR assay. Moreover, although a low level NC-independent incorporation of RNAs in VLPs cannot be excluded, the role of NC on packaging seems to be important for 7SL RNA and not for others Pol-III promoted RNAs such as tRNAs [32].

Finally, in an effort to elucidate the importance of 7SL RNA for APOBEC3G and 3F incorporation into HIV1 virions, we first produced virus from cells in which 7SL RNA was downregulated by RNA interference against SRP14. These particles lacked 7SL RNA but still incorporated other small cellular RNA species such as 5S and Y3, and did not exhibit significant changes in APOBEC3G and APOBEC3F levels. As the decreased infectivity of these virions somewhat hampered the interpretation of our data, we used a second approach by overexpressing SRP19, in order to sequester 7SL RNA away from assembling retroviral particles as recently described [32,40]. The resulting virions exhibited at least ten-fold decreased levels of 7SL RNA, yet no change in their APOBEC3G or APOBEC3F content was detected. Furthermore the infectivity of these 7SL RNA-depleted viruses, whilst normal in the absence of cytidine deaminases, was inhibited at least as effectively as that of control virions by either APOBEC3G or APOBEC3F. We conclude from these data that 7SL RNA is not an essential mediator of APOBEC3G or 3F recruitment into HIV1 virions. Interestingly, a recent study revealed that short (≥ 10 nucleotides) G-rich single-stranded RNA facilitate nucleocapsid-APOBEC3G complex formation in vitro [36]. While this result suggest that a wide range of cellular and viral RNAs could mediate packaging of antiviral cytidine deaminases into HIV1 virions, it would be surprising if a specific RNA species or a specificity-conferring cofactor were not at play in this process, to account for the target specificity of the various antiviral cytidine deaminases.

## Conclusion

The binding of APOBEC3G to 7SL RNA through its Alu domain suggests a mechanism for APOBEC-mediated inhibition of Alu retrotransposition. However, biochemical and functional analyses of ΔVif HIV1 particles devoid of 7SL RNA indicate that this RNA species is not essential for the virion recruitment of the antiviral cytidine deaminase.

## Authors' contributions

DB, SP and DT conceived the overall design of the study, BM performed the initial analysis of the A3G mutants, AL and KS helped with and supervised, respectively, the 7SL RNA binding studies, DB, SP and DT wrote the paper. All authors read and approved the final manuscript.
